# Editorial: Neurotechnology for sensing the brain out of the lab: methods and applications for mobile functional neuroimaging

**DOI:** 10.3389/fnrgo.2024.1454894

**Published:** 2024-08-05

**Authors:** Hasan Ayaz, Frederic Dehais, Giuseppina Pilloni, Leigh Charvet, Marom Bikson

**Affiliations:** ^1^School of Biomedical Engineering Science and Health Systems, Drexel University, Philadelphia, PA, United States; ^2^ISAE-SUPAERO, Université Fédérale de Toulouse, Toulouse, France; ^3^Department of Neurology, NYU Grossman School of Medicine, New York, NY, United States; ^4^Department of Biomedical Engineering, The City College of New York, The City University of New York, New York, NY, United States

**Keywords:** electroencephalography (EEG), functional near-infrared spectroscopy (fNIRS), transcranial direct-current stimulation (tDCS), neuroergonomics, neurotechnology, neuromodulation

With the advent of portable and wearable neuroimaging methods like electroencephalography (EEG), functional near-infrared spectroscopy (fNIRS), and neurostimulation approaches like transcranial direct-current stimulation (tDCS), significant progress has been made in both recording and altering brain activity in real-world contexts outside of the laboratory, in the course of natural environments and unrestricted movement. As a result, these neurotechnologies are now expanding in the clinical field (assistive technologies, motor rehabilitation, etc.), spreading to the entertainment industry to enhance gaming experience, but also extending to the general public through wellbeing (e.g., meditation and relaxation induction, sleep improvement) and domotics (e.g., home automation) applications (Bikson et al., 2020; Charvet et al., 2020; Dehais et al., 2020; Ayaz et al., 2022). Following this trend, the range of neuroergonomics applications that can benefit from neurotechnology broadens. Indeed, they have the potential to alleviate mental and physical loads associated with the repetition of straining actions to improve task performance both in terms of precision and speed and to promote new forms of interactions to enhance human-technology symbiosis in a variety of applications.

This Research Topic invited submissions that are in parallel to the 2022 joint meeting of Neuroergonomics Conference & NYC Neuromodulation Conference and explore recent approaches and emerging directions in neuroergonomics, aiming to advance our understanding of neurophysiological measures and their relationship to complex tasks. The contributions are expected to address a range of topics, focusing on -noninvasive and portable neurotechnologies that can enhance human performance, mitigate disease burden, and deepen our comprehension of complex brain functions.

This Research Topic called for submissions that cover recent approaches and emerging new directions in the development of neurotechnology out-of-the lab and attempted to chart a path toward a better understanding of the neurophysiological measures and improvement of human performance.

Submissions encouraged included advanced neuroscience methods, neuroimaging analysis approaches as well as advanced Artificial Intelligence (AI) and signal processing techniques to decode brain dynamics in actively behaving participants in field settings. Applications of these technologies to investigate cognition, emotion, perception, decision-making, attention, working memory, cognitive workload, performance monitoring, human-machine-interaction, brain-computer interface, mobile brain and body imaging, neuroadaptive technologies and related areas relevant to working environments and health were specially invited.

There were four publications in this Research Topic in addition to the three publications in the parallel Research Topic (Neurotechnology for brain-body performance and health: Insights from the 2022 Neuroergonomics and NYC Neuromodulation Conferences) that utilized wearable brain and body sensors.

The first contribution by Hayne et al. focused on using portable and wearable fNIRS to classify collaborative interactions. The authors aimed to distinguish between cooperative and competitive interactions in pairs of participants playing a decision-making game with uncertain outcomes. They measured fNIRS-based brain activity from social, motor, and executive areas during gameplay, both alone and in competition or cooperation with another participant. The results showed that features from the social areas of the brain outperformed other features in discriminating between competitive, cooperative, and alone conditions during cross-validation. These findings have the potential to enhance human-human teams, by providing targeted feedback in order to improve team outcomes in naturalistic settings using wearable neurotechnology.

The second contribution by Moinnereau et al. studied quantifying time perception during virtual reality (VR) gameplay using a multimodal biosensor-instrumented headset. Time perception is a complex cognitive process that allows individuals to estimate the duration and timing of events. The authors utilized multimodal biosensors embedded in a VR headset capable of measuring EEG, electrooculography (EOG), electrocardiography (ECG), and head movement data while the user is immersed in a virtual environment. The authors extracted features from the biosignals and then mapped them to a predicted time perception rating from the participants. In this report, the authors provide an in-depth analysis and discussion of the top predictive features of biomedical signals. These results can be used to inform the design of more engaging and immersive VR experiences.

The third contribution by Oishi et al. utilized fNIRS to investigate the IKEA effect that refers to a cognitive bias in which the maximum willingness to pay (WTP) for a product is high because the experience of assembling the product is highly valued. In this novel study, participants were asked to perform a WTP evaluation after assembling three types of do-it-yourself (DIY) products and handling three types of Non-DIY products. The cortical activation during the evaluation of WTP for DIY and Non-DIY products exhibited marked differences. The authors concluded that the value of experiential consumption can be assessed using fNIRS-based neuroimaging and provides a novel approach to consumer neuroergonomics. They predicted that visualization of the value of experiential consumption will create marketing opportunities for the industry and will become an indispensable method in the future.

The final contribution by Callan et al. focused on advanced EEG signal processing techniques for removing motion artifacts from brain activity data collected in extremely noisy environments and during demanding tasks. The authors aimed to evaluate whether Artifact Subspace Reconstruction (ASR) and Independent Component Analysis (ICA) can effectively extract brain activity from EEG recordings with substantial artifacts, specifically data recorded while participants were skateboarding on a half-pipe ramp. Their study compared the performance of multiple signal processing pipelines and concluded that these findings affirm the feasibility of recording brain activity during physically demanding tasks involving significant body movement. This research lays the groundwork for future neuroergonomics investigations into the neural processes underlying complex and coordinated body movements.

As the origin of this Research Topic, the 2022 joint meeting of the Neuroergonomics Conference & NYC Neuromodulation Conference was held from July 28 to August 1, 2022, in New York, NY. The following iteration of the Neuroergonomics Conference is scheduled for July 8–12, 2024 in Bordeaux, France, and the following NYC Neuromodulation Conference is scheduled for August 1–3, 2024 in New York City.

## Author contributions

HA: Writing – original draft. FD: Writing – review & editing. GP: Writing – review & editing. LC: Writing – review & editing. MB: Writing – review & editing.
